# Is Dentistry Drifting into Medicine?

**Published:** 1900-07

**Authors:** 


### Editorial.

### IS DENTISTRY DRIFTING INTO MEDICINE?

It has been necessary to occasionally refer to the drift of thought in the dental profession in the direction of a general medical education. The matter has been discussed extensively in various dental bodies, and especially in a series of well-prepared papers read and discussed in the Section of Stomatology, National Medical Association, recently held at Atlantic City, N. J.

If the papers and discussions were confined to those directly connected with dental educational procedure, the results would be of more importance, but, unfortunately, this is not always the case.

There seems to be a tendency in many minds to magnify the importance of the degree of Doctor of Medicine. This may be due to a desire to stand well with the medical profession through the influence supposed to exist in the title, and, possibly, to a very mistaken feeling that the word dentist carries with it some measure of social inferiority. The result of this combined feeling has been a constant agitation among a large class, generally outside of college circles, to effect a change in present educational methods, and to do away with the old degrees in dentistry and substitute therefor the general degree in medicine. It seems to be forgotten that the obliteration of the old titles does not make the possessor of the higher more worthy of recognition from that simple fact. The discussion of this subject has undoubtedly been productive of good results, but it must not be forgotten that all great changes come slowly and cannot be rapidly advanced by theorists, but only through evolutionary changes,—a slow growth from the lower to the higher.

Dental education has been gradually tending towards the medical degree for the past thirty years, and those who have watched the trend of thought have long since come to the conclusion that at no distant period dentistry would be merged into medicine, and that the dentist of the future would practise as a specialist under the one general title of Doctor of Medicine. While this is one of the certainties of the not far distant future, it does not necessarily follow that it will prove satisfactory to those who regard of some value that which has been attained by the dentistry of the past.

It is unnecessary to refer to the cause of the separation of dentistry and medicine. It is usually ascribed, in this country, to the refusal of the medical men of Maryland to accept Dr. Harris's proposition to have established a chair of dentistry in the university, and that this refusal led up to the organization of dental colleges. While it is true this marked an era in dental progress, the real separation dates back to the very beginning of dentistry, lost in the mists of time. The fact that it certainly began as a mechanical art for the replacing of lost dentures settled its status. The professional man could not fraternize with the mechanic, and the latter was equally unfitted to mingle with the former, and thus the gulf widened by the lapse of ages. This continued, with no thought of change, until some dentists, ambitious for a supposed better standing socially and professionally, sought the medical degree, some for the increased information secured in its attainment and others for the better social position it was supposed to give.

During this evolutionary stage the colleges of the higher class have steadily advanced the entrance requirements in this country, and have brought the strictly medical branches very nearly up to the standard demanded of graduates in medicine. Those schools connected directly with universities and the higher medical colleges have the curriculi so arranged that up to a certain limit the dental students are medically educated. In the school with which the writer is connected the curriculum has been steadily advanced for the past twenty years, and has, at present, reached the limit of extension until another year can be added to the present three years course, when it will be still further advanced, leaving nothing to be desired except, it may be, the degree in medicine.

Does this degree carry weight? The answer will be in the affirmative or negative as the individual may view it in all its bearings. To some it means much; to others, who have but little faith in titles, it is not regarded with much respect. It should always mean thorough training and ability to practise medicine, but it is quite evident to those well informed that it only indicates that some have brilliantly answered a series of questions, written or oral, and others have managed to work through by the persistent efforts of the quiz masters. It is, therefore, no sure test that the man who has secured the degree is really qualified to practise his profession. The same applies with equal force to those receiving the dental degree. The second act in this drama of life is the final examination of the State boards, medical and dental, and the farce of final examinations is complete. The truth is as old as humanity that if a man or woman knows a thing, that knowledge is sure to be made apparent, and if he or she continues to occupy a mediocre position, it is equally clear that both are living on the degree. There are dentists who possess a knowledge of medical subjects equal to those medically educated, and there are medical men fairly well informed upon the main facts of dentistry.

There is a tendency in all the discussions of this topic to belittle the work of the dentist. The assumption is that he is following a calling essentially inferior to the older profession. This may not be formulated in words, but it is implied in nearly all the papers of those who advocate the abandonment of the dental degree. "We want a higher standard," is the cry of those outside of college work. Some of those making this demand may know what a higher standard means, but with the majority it is simply a childish request for something not immediately attainable.

Dentistry has no reason to beg for recognition. Such a demand is always an indication of partial culture. Dentistry has a position secured by long years of patient work. It has demonstrated that the oral cavity contains within its borders sufficient to occupy the best thought for a lifetime of those who practise therein. It has proved to the doctors of medicine that for uncounted centuries they have neglected the principal source of disease, and that this neglect, to their discredit, still continues. It has proved that the pathological conditions of this cavity hold intimate relations with the general organization, and are a source of continued infection. It has proved that it has been possible to lessen the terrors of old age and prolong the period of life, and to make that period one of comparative comfort; and, finally, it has forced the respect of the men who treated it in the past with contempt, and they have been obliged, however unwillingly, to believe that the time has come when the partial degrees in dentistry should be abandoned.

What will this mean for dentistry? This brief question contains within it more difficult problems than this article can possibly solve. The answer may be partially outlined by the results of experience. The writer has observed that, in proportion to the advance in the preliminary entrance standard and the curriculum, the technical skill has been lowered. This has been so continuous that it is impossible to ascribe it to some temporary and incidental cause. It is peculiarly marked in those who have attempted the study of dentistry after they have spent the required years in the mastery of medical subjects. In a long experience the writer cannot recall a single instance of a medical man acquiring technical skill during his sojourn at college. The advance of the preliminary entrance standard to the very highest degree possible at the present time—the diploma of a high school, unless that be a high school of manual training—promises to lower the standard of dental attainment. This is no fanciful idea, but is based on actual observation. The results are not in accord with the previous views held by the writer, and are, therefore, in direct opposition to the anticipations held by himself and others. That there are exceptions to be noted remains true, but where these exist the mechanical talent has been developed in earlier years and by other methods.

This statement will not be credited by that class of mind that is constantly demanding a higher standard. Educators of experience accept these changes with hesitation and doubt. They cannot avoid the conclusion that the practical side of dentistry has suffered and will continue to suffer in proportion as the curriculum is extended at the top. Like an inverted pyramid, it must eventually fall by its own weight.

The writer must not be misunderstood. He is not contending for a lower standard; indeed, every desire of his mentality is for a more cultured class in his profession, but it is not what may be desired, but what can be attained.

That we are drifting towards the end most desired by some needs no argument, but when time has solved this problem it will bring with it another for the dentists of that period, and that will be, by what means will they be enabled to recover the then lost art of dentistry?

